# Stimulation of the Defense Mechanisms of Potatoes to a Late Blight Causative Agent When Treated with *Bacillus subtilis* Bacteria and Chitosan Composites with Hydroxycinnamic Acids

**DOI:** 10.3390/microorganisms11081993

**Published:** 2023-08-02

**Authors:** Liubov Yarullina, Ekaterina A. Cherepanova, Guzel F. Burkhanova, Antonina V. Sorokan, Evgenia A. Zaikina, Vyacheslav O. Tsvetkov, Ildar S. Mardanshin, Ildus Y. Fatkullin, Joanna N. Kalatskaja, Ninel A. Yalouskaya, Victoria V. Nikalaichuk

**Affiliations:** 1 Institute of Biochemistry and Genetics, Ufa Federal Research Centre, Russian Academy of Sciences, 450054 Ufa, Russia; k_cherepanova@mail.ru (E.A.C.); guzel_mur@mail.ru (G.F.B.); fourtyanns@googlemail.com (A.V.S.); molgen@anrb.ru (E.A.Z.); zv347@yandex.ru (I.Y.F.); 2 Department of Biology, Ufa University of Science and Technology, 450076 Ufa, Russia; ums@uust.ru; 3 Bashkir Research Institute of Agriculture, Ufa Federal Research Center, Russian Academy of Sciences, 450054 Ufa, Russia; bniishufa@yandex.ru; 4 Institute of Experimental Botany Named after V. F. Kuprevich of the National Academy of Sciences of Belarus, 220072 Minsk, Belarus; nan.botany@yandex.by (J.N.K.); yalouskaya92@mail.ru (N.A.Y.); 5 Institute of New Materials Chemistry, National Academy of Sciences of Belarus, 220141 Minsk, Belarus; ichnm@ichnm.by

**Keywords:** *Solanum tuberosum* L., *Bacillus subtilis*, *Phytophthora infestans*, conjugates, chitosan, caffeic and ferulic acids, pro/antioxidant system, gene expression, PR proteins, induced systemic resistance

## Abstract

*Phytophthora infestans* is, worldwide, one of the main causal agents of epiphytotics in potato plantings. Prevention strategies demand integrated pest management, including modeling of beneficial microbiomes of agroecosystems combining microorganisms and natural products. Chitooligosaccharides and their derivatives have great potential to be used by agrotechnology due to their ability to elicit plant immune reactions. The effect of combining *Bacillus subtilis* 26D and 11VM and conjugates of chitin with hydroxycinnamates on late blight pathogenesis was evaluated. Mechanisms for increasing the resistance of potato plants to *Phytophthora infestans* were associated with the activation of the antioxidant system of plants and an increase in the level of gene transcripts that encode PR proteins: basic protective protein (PR-1), thaumatin-like protein (PR-5), protease inhibitor (PR-6), and peroxidase (PR-9). The revealed activation of the expression of marker genes of systemic acquired resistance and induced systemic resistance under the influence of the combined treatment of plants with *B. subtilis* and conjugates of chitin with hydroxycinnamates indicates that, in this case, the development of protective reactions in potato plants to late blight proceeds synergistically, where *B. subtilis* primes protective genes, and chitosan composites act as a trigger for their expression.

## 1. Introduction

Recently, numerous studies have demonstrated that the use of endophytic bacteria of the genus *Bacillus* can lead to advantageous effects on plant fitness through a broad spectrum of mechanisms, including phytohormone synthesizing and augmenting the availability of minerals to plants [1]. Some of them bring certain benefits, acting as plant-growth-promoting microorganisms (PGPM) [1,2,3]. Endophytic strains of PGPMs which live inside plant tissues without causing damage form the basis of the plant microbiome community. This group is of great interest in terms of the practical application of plant–microbe relationships. Many endophytes are facultative plant symbionts from the genus *Bacillus* [3,4].

*B. subtilis* and *B. thuringiensis* account for more than 75% of the total biopesticides market [5]. The disadvantages of biopesticides include a relatively low rate of eradication of pathogens and high sensitivity to adverse environmental factors [6]. In our opinion, it is very important to increase the stability and diversity of the spectrum of activities of microbiological preparations for plant protection from different biotic and abiotic environmental factors by supplementing the bacterial strain with biologically active substances [2,3,4].

Chitosan and its derivatives are elicitors of plant immunity, which are often recommended to increase the biological activity of biological control agents [1]. It has been demonstrated to inhibit pathogenic microorganisms in vitro by damaging the cell membrane of pathogens and inhibiting synthesis of nucleic acids, leading to cell death [7,8]. These molecules possess the ability to induce plant resistance against a spectrum of invaders and to enhance biodiversity in the rhizosphere [9]. 

The biological activity of chitosan depends on its molecular weight (varies from 10^2^ kDa for oligomers to up to several hundred kDa for high molecular weight forms), degree of polymerization and deacetylation (combination of N-acetylglucosamine and glucosamine residues), concentration, plant species, soil chemistry, and environmental conditions [10]. The highest biological activity is characteristic of chitosan with a degree of deacetylation in the range of 70–90% [11]. Chitooligosaccharides are degradation products of chitosan consisting of depolymerized derivatives of chitosan with a degree of polymerization ranging from 2 to 10 [12]. Chitosan has been shown to be degraded to chitooligosaccharides by chemical methods or enzymatic processes. Many enzymes have been reported to hydrolyze chitosan, such as cellulase, papain, pepsin, pectinase, lipase, and chitosanase (EC. 3.2.1.132), which hydrolyzes chitosan specifically. In recent years, the production of chitosanase by *Bacillus*, *Staphylococcus*, *Microbacterium*, *Streptomyces*, and other bacterial genera has been reported, among which *Streptomyces* and *Bacillus* have provided the greatest numbers of these enzymes [13]. It is suggested that *Bacillus* sp. could be used to efficiently produce active forms of chitosan that are biocontrol agents applicable for safe and sustainable agricultural production.

Thus, *B. velezensis* RC218, in combination with chitosan, demonstrated the ability to reduce Fusarium head blight disease severity and *Fusarium*-derived toxin deoxynivalenol accumulation in *T. aestivum* plants in greenhouse and field trials [14]. In addition, the combination of 0.5% chitin oligomers with *B. subtilis* HS93 or *B. licheniformis* LS674 significantly (by 62% and 70%, respectively) reduced the development of *Phytophthora* and *Rhizoctonia* pepper root rot compared to control plants [15].

Some plant-associated bacteria possess chitinase activity and can be used in the control of pathogens. The expression of chitinase genes in bacteria demands the availability of chitin substrates [16]. Thus, the positive influence of the set of chitinolytic bacterial strains, *Azospirillum lipoferum* and *Pseudomonas fluorescens,* with chitosan on the germinating of *Zea mays* seeds was evaluated. In addition, *P. putida* effectively increased the weight of roots and germinated seeds, but chitosan in combination with *P. putida* increased the weight of the shoots [17]. 

The chemical modification of chitosan makes it possible to obtain derivatives with increased solubility, and antimicrobial, growth-stimulating, and antioxidant activity [18]. The modern vector of chemical modification of chitosan is the inclusion of phenolic compounds in their composition, such as hydroxycinnamic acids, which are precursors of most phenolic compounds and can regulate plant defense responses [19], including those associated with the development of an oxidative burst in response to a pathogenic attack.

Defense reactions against pathogens involve an increase in the production of reactive oxygen species (ROS). H_2_O_2_ can be considered as the most important molecule involved in the transmission of intracellular signals that regulate gene expression and the activity of defense systems [20]. It has been shown that H_2_O_2_ is involved in the activation of the gene expression of stress proteins, including PR1, PR6, PR9, and PR10. However, ROS can cause a variety of damage, such as the inhibition of enzymes, denaturation and inactivation of proteins, and membrane and cytoskeleton injuries [21,22].

Changes in the concentration of H_2_O_2_ in plant tissues during pathogenesis can occur as a result of many metabolic processes, but this primarily occurs as a result of changes in the activity of the antioxidant system, in particular, catalase (EC 1.11.1.6), superoxide dismutase (EC 1.15.1.1.), peroxidase (EC 1.11.1.7), enzymes of the ascorbate–glutathione cycle, proline, etc. [23]. 

Catalase is a common antioxidant enzyme with the highest turnover rate. It is present in living tissues and is a key clinical enzyme involved in the breakdown of hydrogen peroxide to water and molecular oxygen [24]. Catalase activity can be significantly changed by signaling molecules. The effect of H_2_O_2_ on catalase activity in plants is ambiguous. Thus, in wheat seedlings, H_2_O_2_, depending on the concentration, inhibited [25] or stimulated [26] catalase activity. Stress factors can lead to an increase in ROS generation and, as a result, to oxidative injury to plant organisms via inhibition of SOD activity [27]. Peroxidases take part in the strengthening of cell walls due to lignification of cell walls, setting a physical barrier to invaders [28]. It was shown that activation of peroxidase chitinase and phenylalanine ammonia-lyase in tomato plants inoculated with *B. tequilensis* PKDN31 and *B. licheniformis* PKDL10 led to the activation of induced systemic resistance (ISR) against *Fusarium oxysporum*. F. sp. Lycopersici [29]. Activation of the transcription of PR-genes, such as encoding peroxidase (PR-9), endo-1,3(4)-beta-glucanase (PR-2), PR-4, and PR-5 (thaumatin-like protein), was an important mechanism of the biocontrol ability of *B.subtilis* MBI600 [30].

The amino acid proline takes part in the stabilization of plant membranes and the structure of proteins and plays a role in ROS detoxifying [31]. A significant accumulation of proline in plants which are inoculated with endophytic bacteria *B. subtilis* was shown, and its level positively correlated with plant disease resistance [32].

In this regard, the aim of our work was to investigate the effect of chitosan conjugates with caffeic and ferulic acids and their combined preparations with endophytic *B. subtilis* 26D and *B. subtilis* 11VM [33] bacteria on the status of the pro/antioxidant system and changes in the gene expression of pathogen-induced proteins in potato plants infected with the late blight pathogen.

## 2. Materials and Methods

### 2.1. Plant, Microbe, and Insect Material

Plants: Mini-tubers of *Solanum tuberosum* L. cv. Udacha were planted in pots with soil (TerraVita, Nord Pulp) to a depth of 3–4 cm. Plants were propagated in the KBW E6 climatic chamber (Binder GmbH, Tuttlingen, Germany) (16 h light period, 20–22 °C).

PGPM bacteria: Gram-positive aerobic strains *B. subtilis* 26D and *B. subtilis* 11VM were held in the collection of the Laboratory of Biochemistry of Plant Immunity of IBG UFRC RAS [34]. Strains under investigation were grown on liquid LB (lysogeny broth) medium. Bacteria were cultivated at 37 °C using an ES-20 laboratory shaker with an oscillation speed of 120 rpm (Biosan, Tarnėnai, Lithuania). Culture growth was measured spectrophotometrically (Mini-SpecBio, BioRad, Hercules, CA, USA) at 590 nm and expressed as optical density units (OD590).

“Plant+endophyte” System: 15-day-old plants were inoculated according to the previously described method [35] with 5 mL of (1) water (control plants); (2) solutions of conjugates of chitosan with caffeic (ChCA, 0.30 mg/mL) or ferulic (ChFA, 0.25 mg/mL) acids; (3) suspensions of bacteria *B. subtilis* strains 26D or 11 VM (10^8^ cells/mL); or (4) a mixture of ChCA or ChFA solutions with bacterial suspensions of the corresponding strains (compositions for treatment were 0.30 mg/mL ChCA+10^8^ cells/mL and 0.25 mg/mL ChFA+10^8^ cells/mL of bacterial strains).

Pathogen and infection of plants: Zoospores of the oomycete *P. infestans* 1840 were used to infect potato plants. It was re-inoculated from infected potato mini-tubers to restore aggressiveness and grown on potato glucose agar for 7 days. The surfaces of colonies were washed with distilled water at 4 °C for 30 min. The concentration of sporangia was evaluated in a Fuchs-Rosenthal chamber. Plants were infected with 5 mL per plant of a 1 × 10^5^ spores/mL suspension on the 3rd day after treatment with chitosan conjugates and inoculation with the *Bacillus* suspension. On the 10th day after infection of plants with the late blight pathogen, the disease severity was measured by the percentage of the lesioned area to the total area of the leaves. Images were examined with the ImageJ software ver. 1.54a (NIH, Bethesda, MD, USA). Plants treated with water and not infected with phytophthora were used as a control.

### 2.2. Obtaining Conjugates of Chitosan with Hydroxycinnamic Acids

The conjugates of chitosan with CA and FA were obtained by the carbodiimide method according to the procedure described in [36]. For the synthesis of conjugates, we used 30 kDa oligomers of chitosan (degree of deacetylation 98.3%, degree of polymerization ~186) manufactured by Glentham Life Sciences (Corsham, UK), CA (M = 180.16 g/mol, Sigma-Aldrich, Burlington, MA, USA), FA (M = 194,18 g/mol, Sigma-Aldrich, USA), and 1-ethyl-3-(3-dimethylaminopropyl)carbodiimide hydrochloride (EDC, Sigma-Aldrich). The conjugate was synthesized at a mass ratio of ChCA/ChFA = 5:1; EDC was taken in a threefold molar excess with respect to CA and FA. The content of CA and FA in the synthesized conjugates was determined spectrophotometrically (Specord-50, Jena, Germany). The absorption spectrum of the conjugate was recorded in the range of 200–400 nm, and the content of CA and FA was calculated using a preliminarily built calibration graph. The degree of attachment of the caffeic and ferulic acids to the chitosan was 5.0 ± 0.6% or 53.8 ± 7.2 μg/mg of chitosan.

### 2.3. Hydrogen Peroxide Content Measurement

Leaves (200 mg fresh weight) were homogenized using a mortar and pestle in 600 μL of 25 mM sodium phosphate buffer (PB) with a pH of 6.2. The homogenates were centrifuged for 10 min at 13,000× *g* in a 5415R centrifuge (Eppendorf, Ulm, Germany). The H_2_O_2_ production in plants was measured using xylenol orange test [37] with the following reagents: reagent 1: 0.074% Mohr salt (Fe_2_(NH_4_)2SO_4_, purity 99.997%) diluted with 5.81% H_2_SO_4_; reagent 2: 0.009% xylenol orange diluted by 1.82% sorbite. Reagent 1 was mixed with reagent 2 at a ratio of 1:100; a total of 25 μL of homogenized and centrifuged sample was added to 250 μL of reaction mixture. The ferric product–xylenol orange complex optical density (OD) was detected spectrophotometrically at 560 nm (EnSpire plate reader; Perkin Elmer, Waltham, MA, USA). The concentration of H_2_O_2_ was defined using the calibration curve.

### 2.4. SOD Activity 

Activity of the enzyme was evaluated using the previously described method [38]. After homogenization in 50 mM of TrisHCl buffer (pH 7.4), plant extracts were centrifuged for 10 min at 10,000× *g* and 4 °C in a 5415R centrifuge (Eppendorf, Germany), and 100 µL of supernatants were added to the mixture of reagents as described in [38]. Optical density was immediately measured (OD0) in a LS 55 luminescence spectrometric cell (Perkin Elmer, USA) at 540 nm. Then, after 10 min, a second measurement was taken (OD10). The absorbance change was used to calculate the percentage of the nitro blue tetrazolium reduction: U = (OD10 − OD0)/D0 × 100% was taken as an activity unit. SOD activity was expressed in units/mg protein min.

### 2.5. Peroxidase (PO) and Catalase (CAT) Activity

Plant leaves were homogenized with mortar and pestle in 50 mM of Na-phosphate buffer with a pH of 6.2 (1:5). After incubation (30 min at 4 °C), extracts were centrifuged at 13,000× *g* for 15 min (5415 K; Eppendorf, Hamburg, Germany). A number of 96-well plates (Corning-Costar, Glendale, AZ, USA) were used for the measures. Optical density was measured on the EnSpire plate reader (Perkin Elmer, USA) spectrophotometer. PO activity was accessed by the OD of oxidased (o^−^) phenylenediamine in the presence of H_2_O_2_ at 490 nm [39]. The enzyme activity was expressed in OD/mg of protein minute. CAT activity was assessed based on the ability of H_2_O_2_ to form a stable-colored complex with molybdate salts (OD 405 nm) [40]. Catalase activity was calculated using the formula U = (Ac − Ae)/(KVT) (Ac and Ae are the absorption of the control (containing water instead of the sample) and experimental samples, respectively; V is the sample volume, 0.1 mL; T is the incubation time, 600 s; and K is the coefficient of H_2_O_2_ molar absorption equal to 22.2 × 10^3^ mol^−1^ cm^−1^). CAT activity was expressed as units/mg of protein min. Protein content was determined by the Bradford method [41].

### 2.6. Native Electrophoresis of PO

To investigate the isoenzyme spectrum of PO, 30 µL of plant extracts (60 µg of protein in 0.05 M PB, pH 6.2) were applied to a 10% polyacrylamide gel (PAAG) plate (1.5 mm thick) and prepared in a buffer containing 50 mM TRIS-HCl, pH = 6.8; 0.3 M sucrose; 0.02% sodium ascorbate; 2 mM MgCl_2_; and 1 mM PMSF.

Electrophoresis in PAAG was carried out for 4 h in two modes: at 10 mA, 40 V before entering the separating gel and at 20 mA, 140 V in the separating gel on the SE-250 mini-electrophoresis device (Amersham Bioscienses, Buckinghamshire, UK). 

The isoenzyme spectrum of the plant PO was determined after isoelectrofocusing (IEF) of protein extracts (60 µg of protein in 0.05 M PB, pH 6.2) on a Hiiu-Kallur electroforetic cell (Estonia) using 7% polyacrylamide gel (PAAG) and 2.5% ampholytes (BioRad, USA) with a pH range of 3.0–10.0. The gel, after electrophoresis, was immersed in a 0.05% solution of diaminobenzidine 0.1 M PB with 0.47 μM H_2_O_2_ until the appearance of brown coloration on the bands of PO isoenzymes. After the appearance of bands, the PAAG was scanned using a HPScanjet G405 device (HP, Shaanxi, China). Identification of the isoelectric point (*pI*) of potato PO was performed using the IEF Standards kit with a *pI* range of 4.45–9.6 (BioRad, USA). Marker proteins were stained with Coomassie Blue. Images were analyzed using the ImageJ program (NIH, USA).

### 2.7. Proline Content

Potato leaves (250 mg) were immersed in 2.5 mL of distilled water and processed according to [35]. The optical density of the reaction products was measured on EnSpire equipment (Perkin Elmer, USA) at 522 nm.

### 2.8. RNA Extraction and Real-Time PCR (qPCR) 

Total RNA was isolated from plants with Lira^®^ reagent (Biolabmix, Novosibirsk, 630090 Russia) according to the manufacturer’s protocol on the 3rd day post infection. Synthesizing of first strand cDNA and the real-time procedure were performed as described previously [35]. The expression of genes was shown as a fold change normalized to the transcription of the reference gene StAct encoding potato actin. iCycler iQ5 Real-Time Detection System 181 equipment and software ver. 2.1 (BioRad, USA) were used. The primers used for qPCR are shown in Table 1. The efficiency of primers was accessed using a 10-fold cDNA dilution series.

### 2.9. Statistical Analysis

There was established five biological and three technical repetitions of experiments. Data presented are mean values with standard errors (±SE). Asterisks on figures’ labels indicate significant differences between treatments and the control (treated with distilled water, non-inoculated and non-infected plants) according to the Kruskal–Wallis test. In order to assess the statistical significance of the differences among some biological groups, Duncan’s test was performed (Supplementary Material). The software Statistica 12.0 (Stat Soft, Moscow, Russia) was applied.

## 3. Results

### 3.1. Susceptibility of Leaves to P. infestans 

**Figure 1:**
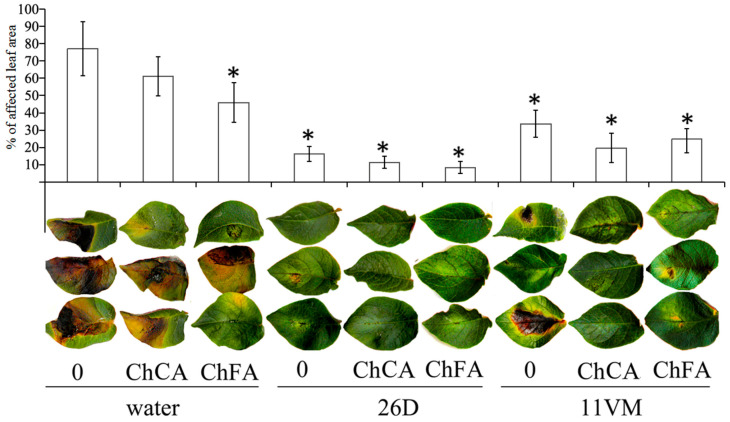
The influence of conjugates of chitin oligomers with caffeic (ChCA) and ferulic (ChFA) acids in compositions with *B. subtilis* 26D and *B. subtilis* 11VM on late blight disease symptoms on potato leaves on the 10th day after *P. infestans* inoculation. Values marked by an asterisk are significantly different from the control as measured by the Kruskal–Wallis test.

The degree of susceptibility was measured by the percentage of leaf area damaged (Figure 1). ChCA had no effect on this parameter. Treatment of plants with ChFA, *B. subtilis* strains, and combinations of *B. subtilis* + ChCA or ChFA resulted in increased resistance to *P. infestans*, with lower percentages of leaf area damaged than the non-treated plants. Individual treatment of plants with ChFA and *B. subtilis* 11VM resulted in 30% and 40% decreases in the lesioned area, respectively. *B. subtilis* 26D treatment reduced lesion margins on leaves to a greater degree, and the addition of chitin hydroxycinnamates, in particular ChFA, significantly increased *B. subtilis* 26D effectiveness. Treatment with ChCA and ChFA combined with inoculation by *B. subtilis* 11VM reduced susceptibility to *P. infestans* in comparison to the control and individual ChFA treatment, but treated plants showed high leaf area damage (24.3% and 35.2%, respectively) compared to plants inoculated with *B. subtilis* 26D (Figure 1).

### 3.2. Content of Proline and H_2_O_2_

**Figure 2:**
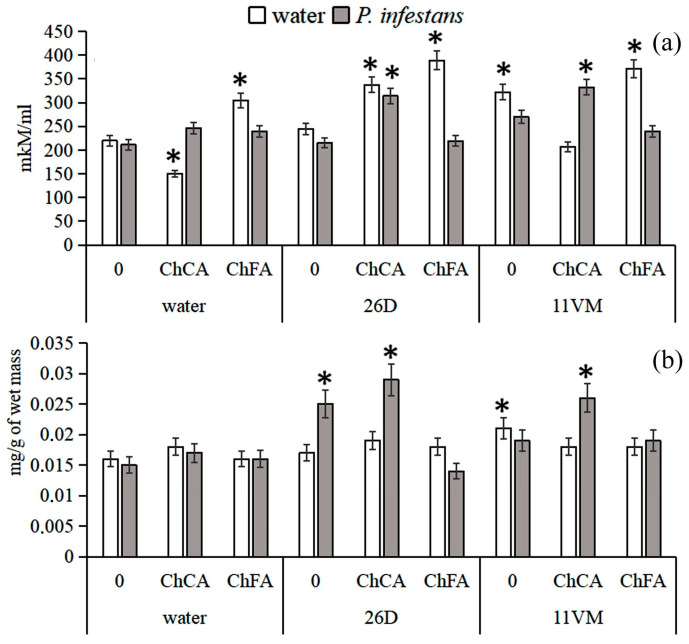
The influence of conjugates of chitin oligomers with caffeic (ChCA) and ferulic (ChFA) acids in compositions with *B. subtilis* 26D and *B. subtilis* 11VM on the content of H_2_O_2_ (**a**) and proline (**b**) in potato plants on the 3rd day after *P. infestans* inoculation. Values marked by an asterisk are significantly different from the control as measured by the Kruskal–Wallis test.

Figure 2 shows that ChFA promoted high levels of H_2_O_2_ individually and in both combinations. Infection of ChFA-treated (including ChFA + *B. subtils* 26D or ChFA + *B. subtilis* 11VM) plants did not stimulate these levels. Under the influence of ChCA, a significant decrease in H_2_O_2_ levels was observed in *B. subtilis* non-infected and non-inoculated plants, but in infected plants, this parameter was at least a quarter higher than in the control plants. Treatment of plants with *B. subtilis* 26D individually did not lead to increase in H_2_O_2_ content, but *B. subtilis* 11VM promoted it (to the same degree as ChCA) in non-infected plants.

*P. infestans* damage did not change the level of proline in potato plants within 3 days after inoculation (Figure 2). The level of proline was significantly increased in infected plants treated with *B. subtilis* 26D and *B. subtilis* 11VM + ChCA, but the highest degree of increase was seen in plants treated with the combination of *B. subtilis* 26D with ChCA. 

### 3.3. Activity of Antioxidant Enzymes

**Figure 3:**
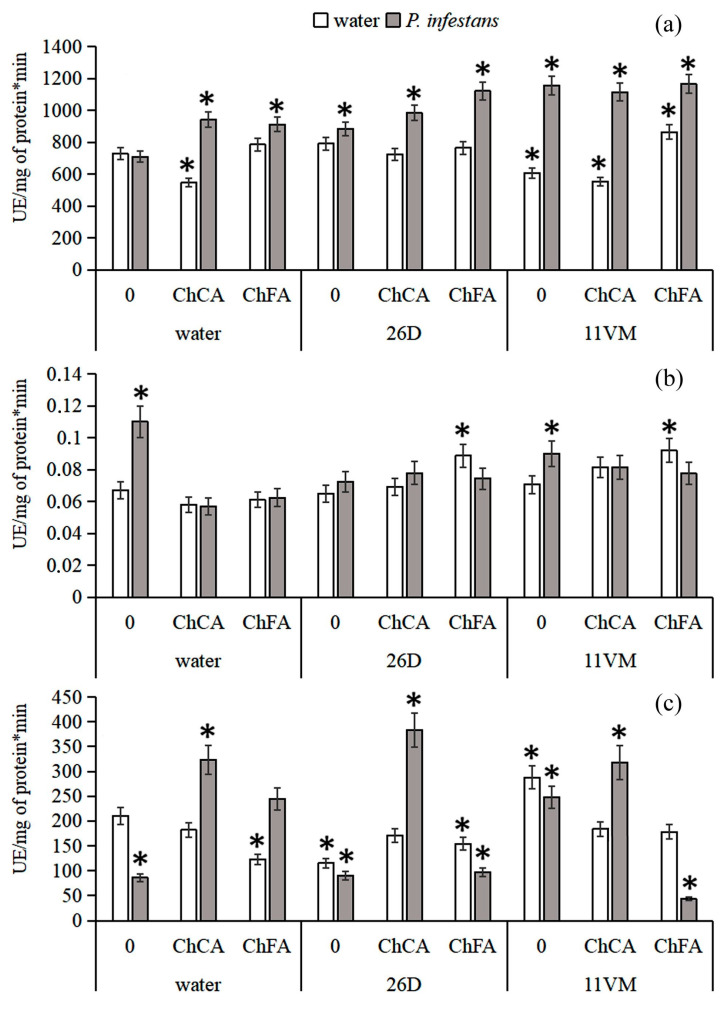
The influence of conjugates of chitin oligomers with caffeic (ChCA) and ferulic (ChFA) acids in compositions with *B. subtilis* 26D and *B. subtilis* 11VM on the activity of peroxidase (**a**), SOD (**b**), and catalase (**c**) in potato plants on the 3rd day after *P. infestans* inoculation. Values marked by an asterisk are significantly different from the control as measured by the Kruskal–Wallis test.

Figure 3a shows that treatment with ChFA and ChCA + *B. subtilis* 11VM led to decreased levels of PO activity in approximately 20–25% of all non-infected plants as compared with control ones. Infection of plants treated with ChFA, ChCA, and *B. subtilis* 26D increased these levels by 25%; all other combinations under investigation produced more significant effects and led to an increase in this parameter by 50% as compared with the control. The maximal level of SOD activity was observed in non-treated plants infected with *P. infestans* (60% higher than in the control) (Figure 3b). Combined treatment of plants with both *B. subtilis* strains and ChFA led to the increase in this parameter in 35% of non-infected plants, but only under the individual influence of *B. subtilis* 11VM was a 30% increase in SOD activity demonstrated in the infected ones. In contrast, the activity of catalase in non-treated plants consisted of less than one-half of the parameter in non-infected and non-treated plants (Figure 3c). It was found that in non-infected plants only *B. subtilis* 11VM promoted catalase activity. Under the influence of ChFA and *B. subtilis* 26D individually and in combination, activity of the enzyme was lower than in the control ones by 30–40%, and catalase activity decreasing was more significant under *P. infestans* inoculation.

### 3.4. Activity of PO Isoenzymes

**Figure 4:**
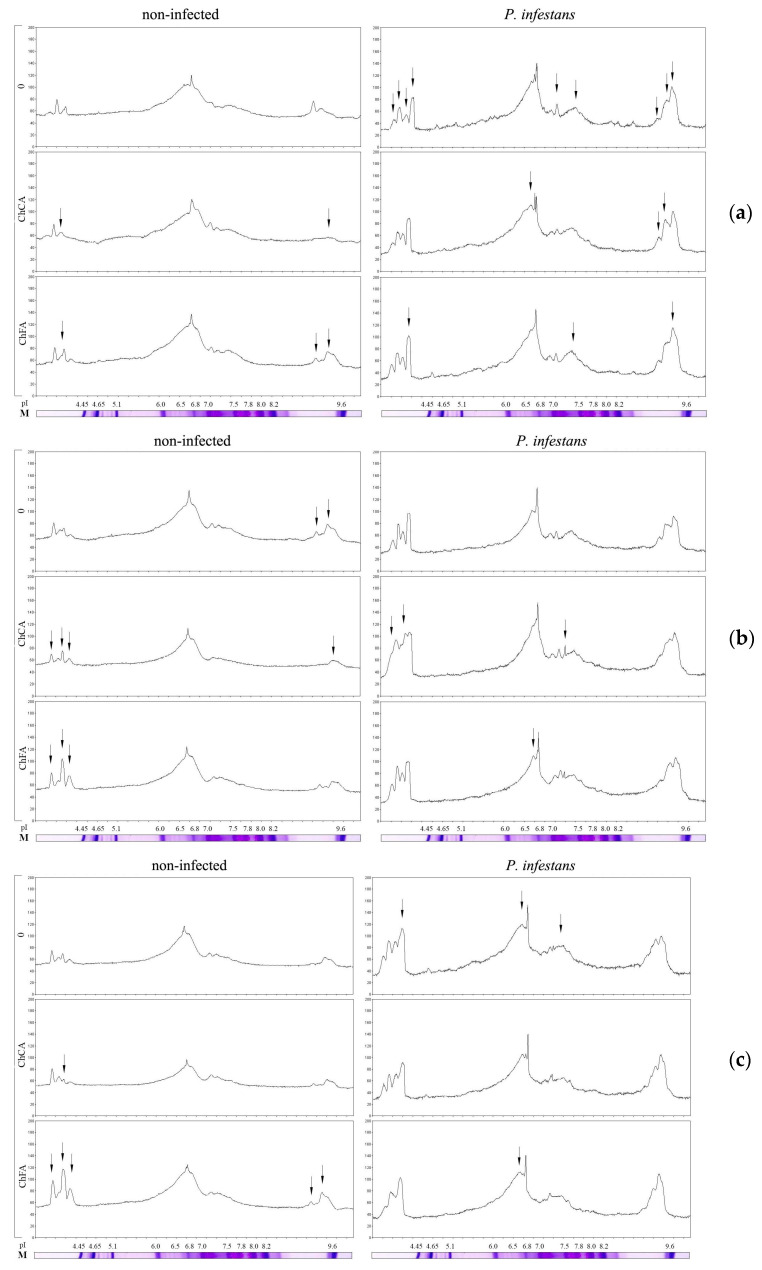
The influence of conjugates of chitin oligomers with caffeic (ChCA) and ferulic (ChFA) acids in compositions with water (**a**), *B. subtilis* 26D (**b**), and *B. subtilis* 11VM (**c**) on the activity of different isoforms of peroxidase on the 3rd day after *P. infestans* inoculation. Arrows indicate important peaks on densitograms.

It is shown (Figure 4) that in ChCA-treated uninfected potato plants, the activity of cationic isoperoxidases, especially *pI*~9.0 and one of the anionic isoforms (*pI*~4.1) of peroxidase, that is constitutively present in untreated uninfected plants, significantly decreased. Treatment of plants with ChFA or *B. subtilis* 26D led to significant increase in the activity of anionic isoperoxidases with *pI*~3.7–4.3 and cationic isoperoxidases with *pI*~9.0–9.3, as well as isoforms in the neutral region (with *pI*~7.1–7.4), compared to untreated controls. Treatment with *B. subtilis* 11VM stimulated the activity of isoperoxidases to a lesser extent compared with *B. subtilis* 26D. Combined treatment of *B. subtilis* 11VM + ChCA did not influence the pool of potato isoperoxidases. In contrast, *B. subtilis* 11VM + ChFA markedly increased the activity of most isoperoxidases under investigation, especially the activity of isoforms with *pI*~4.1 and *pI*~9.3. 

In infected plants, the activity of anionic isoperoxidases with *pI*~3.7–4.3 and cationic isoperoxidases with *pI*~9.0–9.3, as well as isoforms in the neutral region (*pI*~7.1–7.4), increased significantly. The intensity of this pattern was the most pronounced in plants treated with *B. subtilis* 26D + ChCA. Thus, isoperoxidases, which activate upon infection, increased their activity in uninfected plants treated with endophytic bacteria in combination with ChFA composites and in infected plants inoculated with both bacterial strains individually and in combination with chitin hydroxycinnamates.

### 3.5. Transcriptional Activity of PR Genes 

**Figure 5:**
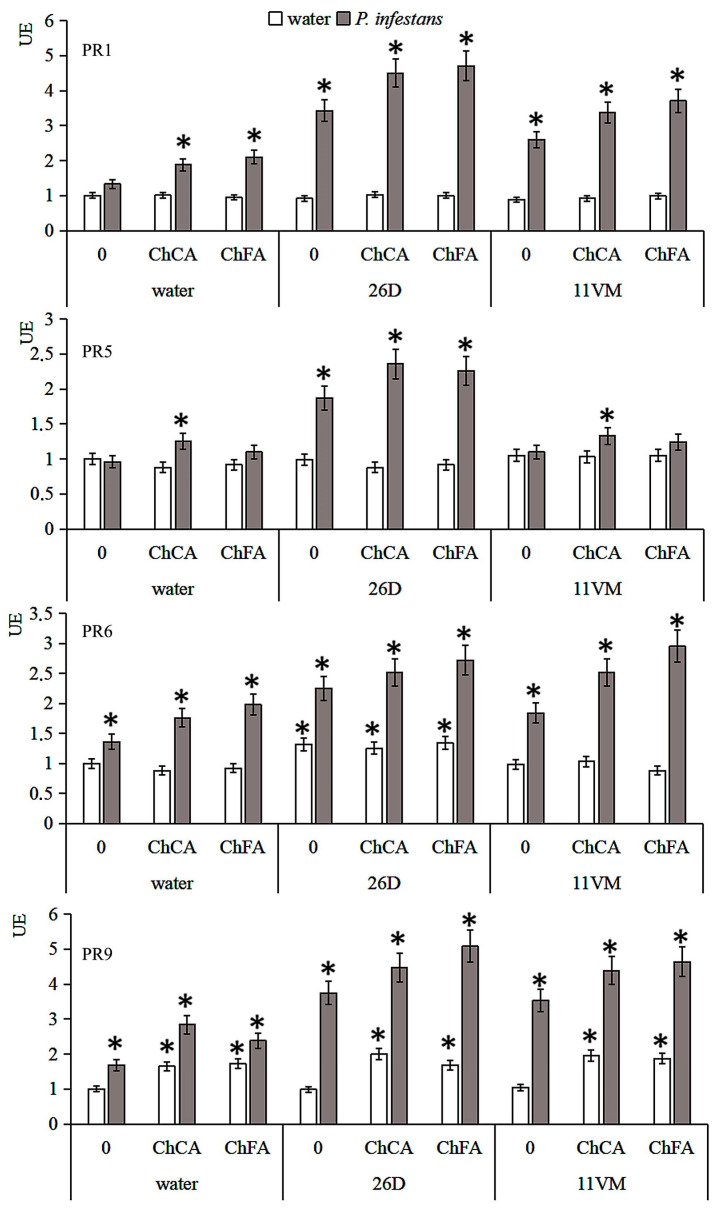
The influence of conjugates of chitin oligomers with caffeic (ChCA) and ferulic (ChFA) acids in compositions with *B. subtilis* 26D and *B. subtilis* 11VM on the content of potato gene transcripts encoding PR1, PR5, PR6, and PR9 proteins on the 3rd day after *P. infestans* inoculation. Expression values were normalized to the housekeeping gene as an internal reference and expressed relative to the normalized expression levels in control plants. Values marked by an asterisk are significantly different from the control as measured by the Kruskal–Wallis test.

The influence of *Bacillus* and conjugates of chitin with hydroxycinnamates on the level of gene transcripts of *StPR1*, *StPR5*, *StPR6*, and *StPR9*, encoding the protein *PR*-1 (marker of the salicylate-dependent pathway), *PR*-5 (thaumatin-like), *PR*-6 (proteinase inhibitor, marker of the jasmonate-dependent pathway) and *PR*-9 (peroxidase), respectively, (Table 1) in intact and *P. infestans* infected plants was investigated. Treatment of potato seeds with cells of *Bacillus* spp. Strains did not influence the transcript levels of the genes under study in non-infected plants (Figure 5). The transcript level of the *StPR9* gene, which encodes anionic peroxidase, unlike *StPR1* and *StPR6,* was slightly upregulated by *P. infestans* inoculation. Treatments of potato plants with conjugates of chitin with hydroxycinnamates increased the transcript levels of the *StPR1*, *StPR6,* and *StPR9* genes and did not alter *StPR5* gene transcription in plant leaves infected with *P. infestans*. 

*Bacillus* sp., especially *B. subtilis* 26D, stimulated transcription of *StPR1* and *StPR6* two-fold and *StPR9* four-fold in infected plants. The *StPR5* gene increased in infected Bs26D-treated plants, but did not in Bs11VM-treated plants. Treatments of plants with *B. subtilis* 26D strain cells and conjugates of chitin with hydroxycinnamates significantly increased the transcript levels of *StPR1*, *StPR5*, *StPR6,* and *StPR9*. Conversely, infecting plants treated with *B. subtilis* 11VM strain and conjugates of chitin with hydroxycinnamates with cells of *P. infestans* led to the increase in transcript levels of *StPR1*, *StPR6,* and *StPR9*, but did not influence *StPR5* transcription.

## 4. Discussion

It was shown that the area of lesions on plant leaves caused by infection with the oomycete *P. infestans* decreased when plants were treated with composites of chitosan with hydroxycinnamic acids, as well as their mixtures with *B. subtilis* bacteria, especially in plants treated with *B. subtilis* 26D + ChFA (Figure 1).

The generation of ROS (superoxide O_2_^−^, hydroxyl OH·, and H_2_O_2_) and cascade of subsequent defense reactions is the earliest response of the plant organism to pathogen attacks. At the same time, ROS accumulated in affected plants causes injury to the organism.

In our studies, in healthy plants treated with the ChCA composite, both separately and in a mixture with *B. subtilis* 26D and 11VM strains, the content of hydrogen peroxide exceeded the level of control plants (Figure 2). An increased level of H_2_O_2_ was also detected in infected plants treated with a complex of bacteria with a ChCA composite. This could be due to the protective effect of bacterial metabolites. It is known that under the action of *B. subtilis*, the activity of antioxidant enzymes and the level of proline in plants can increase [42]. H_2_O_2_ is involved in triggering the hyper-sensitive reaction and lignification processes and may also exhibit antimicrobial activity [43]. It is demonstrated that potato resistance to the late blight pathogen strictly demands the mechanisms related to H_2_O_2_ action [44].

Chitin, chitosan, and their oligomers are active elicitors of plant immunity, including through the regulation of the redox status of plant cells [45,46]. It is known that hydroxycinnamic acids have pronounced antioxidant properties, reducing the formation of reactive oxygen species by neutralizing free radicals [47] and activating antioxidant enzymes [48]. Under the influence of stress factors of different natures, hydroxycinnamic acids can be included in the phenylpropanoid pathway, changing the direction of the synthesis of their own derivatives [49] and generally enhancing the formation of phenolic compounds involved in the mechanisms of increasing plant resistance [50]. 

H_2_O_2_ can be considered as the most important molecule involved in the transmission of intracellular signals that regulate gene expression and the activity of defense systems [44], including increases in the concentration of calcium ions in the cytosol, which play an important role in the transmission of signaling information to the plant genome. It has been shown that H_2_O_2_ is involved in the activation of the expression of stress protein genes [51]. The combination of *B. subtilis* bacteria and chitosan composites with hydroxycinnamic acids has a modulating effect on the generation of ROS and facilitates the rapid transmission of signals that trigger other defense mechanisms to prevent the development of the pathogenic invasion.

Our investigations have revealed an antistress effect of the treatment with *B. subtilis* combined with ChCA on potato plants infected with *P. infestans* (Figure 2). Proline is considered as an important antistress metabolite, which can regulate intracellular processes. In addition, proline takes part in systemic plant defense reactions as a signaling molecule during the attacking of plants by different pathogens [31]. In our work, inoculation of plants with both endophytic strains of *B. subtilis* led to the accumulation of proline [32].

Proline synthesis can be induced under the influence of exogenic signaling mediators salicylic acid [52], brassinosteroids [53], and chitosan [54]. The increase in the content of proline in the leaves of infected potato plants, revealed in this study, is probably associated with both the elicitor nature of chitosan and the action of hydroxycinnamic acids [55]. It is known that increased proline synthesis and activation of its transport between cell compartments and plant tissues is one of the primary and universal plant responses to stress [56]. Changes in the concentration of H_2_O_2_ in plant tissues during pathogenesis can occur as a result of many metabolic processes, but to a greater extent, this occurs as a result of changes in the activity of antioxidant enzymes [57].

In infected plants, we observed an increase in SOD activity (Figure 3); however, treatment with both *Bacillus* strains + ChCA/ChFA decreased SOD activity in the affected ones. It is known that the intensity and the time of exposure to stress factors can modify SOD activity in various manners [58]. Thus, oxidative damage to plant cells and tissues can be a result of a decrease in SOD activity under severe stress influence [27]. Consequently, the utilization of ROS in infected plants is determined by a broad spectrum of other enzymes (catalases, peroxidases, and enzymes of the ascorbate–glutathione cycle) [59]. 

In our work, in potato plants infected with *P. infestans*, a decrease in catalase activity was observed compared to the control (Figure 3). However, in plants treated with composites of ChCA and ChFA, as well as in their combination with bacteria, catalase activity increased several times. Catalase activity can be significantly modified with the participation of signaling molecules. H_2_O_2_ is not only a signaling molecule, but also a catalase substrate. At the same time, its effect on catalase activity in plants is ambiguous. Thus, in wheat seedlings, H_2_O_2_ can inhibit [25] or stimulate [26] catalase activity depending on its concentration.

PO (*PR*-9 family) activity involves both the generation and utilization of H_2_O_2_ [60]. It is involved in the strengthening of cell walls due to the polymerization of phenolic compounds into lignin in cell walls with the participation of H_2_O_2_. It was shown that when plants were infected with the late blight pathogen, a significant activation of PO was observed in all variants of treatment, most significantly when both bacterial strains were combined with ChFA (Figure 3). The regulation of H_2_O_2_ content in potato plants under the influence of both strains of *B. subtilis* with ChCA and ChFA under *P. infestans* infection can likely occur in various ways: through a decrease in SOD activity, an increase in catalase and PO activity, the regulation of the balance of synthesis/inactivation of ROS, and the stabilization of plant metabolism. These parameters can be crucial factors for maintaining plant resistance to environmental fluctuations [61].

Plants produce a wide range of PO isoforms sensitive to various impacts [62]. For example, anionic peroxidases are involved in the formation of resistance to hypo- and hyperthermia, changes in the pH of the environment, and pathogen attacks [63,64].

Analysis of the isoenzyme spectrum of the soluble fraction of peroxidase showed that infection increased the activity of anionic POs with *pI* ~3.7 and 4.3 and cationic isoPOs with *pI* ~9.0–9.3 (Figure 5). It is interesting that the same isoPOs were activated during the joint treatment with *B. subtilis* 26D bacteria with the ChFA composite as during infection with *P. infestans*. It has been shown that cationic isoperoxidases, which have an affinity for the cell walls of the oomycete *P. infestans*, occupy an important place in potato responses to infection with late blight pathogen [40]. The decrease in the lesion area under *P. infestans* on potato leaves treated with *B. subtilis* 26D + ChFA may be directly related to the primed status of the plant. This set makes it possible to implement stronger and faster defense reactions against subsequent pathogen invasion, which manifests itself as a common feature of systemic resistance induced by beneficial microorganisms [65].

Plant treatment with the strains under investigation promoted the development of jasmonate-mediated ISR, as could be identified by the high rate of expression of the *PR*-6 gene, which is considered as marker of ISR in plants [66]. It is worth noting that the development of SAR occurs when a plant recognizes a biotrophic pathogen or its elicitors, which leads to the synthesis of salicylate-dependent PR proteins [67], including the expression of the *PR*-1 gene (marker of SAR) [68].

It should be noted that in infected plants pretreated with *B. subtilis* 26D and 11VM together with ChCA and CaFA composites, a high level of transcriptional activity of the main antimicrobial protein *PR*-1 and the protease inhibitor *PR*-6 genes was observed. It is possible that in these plants, salicylate- and jasmonate-dependent pathways occurred simultaneously. In infected plants treated with *B. subtilis* 26D in combination with ChFA, the transcriptional activity of the thaumatin-like protein (*PR-5*) and PO (*PR-9*) genes significantly increased. It is believed that the activity of proteins of the *PR*-5 family is associated with an increase in membrane permeability [69]. Infected plants treated with both *B. subtilis* strains under investigation in combination with ChFA significantly increased their expression of the *PR-9* gene, which correlates with their resistance. Thus, the induction of plant resistance mediated by the *Bacillus* strains under consideration can be characterized by a multifaceted priming process, including the expression of *PR* genes and fine-tuning regulation of the redox status of plants [70]. 

## 5. Conclusions

Thus, joint treatment of plants with bacterial strains *B. subtilis* 26D and 11VM with conjugates of chitosan with caffeic and ferulic acids led to an increase in potato plants’ resistance to the late blight pathogen. This status involved the activation of CAT, PO, and SOD, accumulation of H_2_O_2_, and promotion of the expression of genes encoding the *PR-1*, *PR-5*, *PR-6*, and *PR-9* proteins. The revealed activation of the expression of the *PR-1* (systemic acquired resistance marker) and *PR-6* (induced systemic resistance marker) genes in plants treated with endophytic *B. subtilis* 26D and 11VM and chitosan composites with hydroxycinnamates indicates that, in these cases, the different branches of protective reactions in potato plants against the pathogen proceed synergistically. It is possible that endophytes prime plant defense genes, and chitosan composites initiate their expression. The mechanism of the influence of ChCA and ChFA on the formation of resistance to the pathogen is determined by the composition (polymer–antioxidant) and the structure of the conjugate itself. Due to the polymer matrix, a prolonged effect of hydroxycinnamic acids on the functional state of the plant cell is probably provided. 

## Figures and Tables

**Table 1:** Primers used in PCR for investigation of the activity of genes under study.

Gene Products	Genes	GenBank Accession Number	Sequence (5′-3′)	
			Forward Primers	Reverse Primer
Actin	StAct	X55749	gat-ggt-gtc-agc-cac-ac	att-cca-gca-gct-tcc-att-cc
PR1	StPR1	AY050221	tgg-gtg-gtg-gtt-cat-ttc-ttg-t	cat-tta-att-cct-tac-aca-tca-taa-g
Thaumatin-like, PR5	StPR5	AY737317	ccc-gtt-tga-cat-tga-cct-ttg	cga-ata-cgg-tgg-aac-atg-ga
Proteinase inhibitor, PR6	StPR6	JX683427	ggg-aaa-gaa-tat-gct-caa-gtt-at	aat-tct-cca-tca-tct-tcc-act-g
Peroxidase, PR9	StPR9	M21334	gta-atc-ctg-ccg-cac-aac-t	gca-gca-aaa-tct-cca-agg-aa

## Data Availability

Not applicable.

## Supplementary Materials

The following supporting information can be downloaded at: https://www.mdpi.com/article/10.3390/microorganisms11081993/s1, Table S1: The experimental groups, demonstrating significant differences by Duncan’s test.
