# Supplementary material for: Stimulation of the Defense Mechanisms of Potatoes to a Late Blight Causative Agent When Treated with Bacillus subtilis Bacteria and Chitosan Composites with Hydroxycinnamic Acids
Source: Microorganisms. 2023 Aug 2;11(8):1993. doi: 10.3390/microorganisms11081993 (PMC10458051; doi:10.3390/microorganisms11081993)
Supplement: Supplementary file 1 [file microorganisms-11-01993-s001.zip › microorganisms-2505841-supplementary.pdf]

Table S1

The experimental groups, demonstrating significant differences by the Duncan's test.

**Parameter**	**Experimental group 1**	**Experimental group 2**	**p-value**
Symptoms	Water + ChFA	26D	0,01
Symptoms	Water + ChFA	26D + ChFA	0,02
Symptoms	26D + ChFA	11VM	0,03
Symptoms	26D + ChFA	11VM + ChCA	0,04
H_2O_2	Water + ChCA	Water + ChFA	0,01
H_2O_2	Water + ChCA	26D + ChCA	0,001
H_2O_2	Water + ChCA	26D + ChFA	0,001
H_2O_2	Water + ChCA	11VM	0,001
H_2O_2	Water + ChCA	11VM + ChFA	0,001
H_2O_2	26D	26D + ChFA	0,001
H_2O_2	26D	11VM + ChFA	0,001
H_2O_2	11VM + ChCA	11VM + ChFA	0,001
H_2O_2	26D + *P. infestans*	11VM + ChCA + *P. infestans*	0,02
H_2O_2	26D + ChFA + *P. infestans*	11VM + ChCA + *P. infestans*	0,03
Proline	26D + ChCA + *P. infestans*	26D + ChFA + *P. infestans*	0,01
Proline	26D + ChFA + *P. infestans*	11VM + ChCA + *P. infestans*	0,03
Peroxidase	11VM + ChCA	11VM + ChFA	0,04
Catalase	Water + ChFA	11VM	0,01
Catalase	26D	11VM	0,01
Catalase	Water + *P. infestans*	Water + ChCA + *P. infestans*	0,01
Catalase	Water + *P. infestans*	26D + ChCA + *P. infestans*	0,001
Catalase	Water + *P. infestans*	11VM + ChCA + *P. infestans*	0,01
Catalase	26D + *P. infestans*	26D + ChCA + *P. infestans*	0,01
Catalase	26D + ChFA + *P. infestans*	26D + ChCA + *P. infestans*	0,02
Catalase	Water + ChCA + *P. infestans*	11VM + ChFA + *P. infestans*	0,01
Catalase	26D + ChCA + *P. infestans*	11VM + ChFA + *P. infestans*	0,01
Catalase	11VM + ChCA + *P. infestans*	11VM + ChFA + *P. infestans*	0,01
